# Stress fibers, autophagy and necrosis by persistent exposure to PM_2.5_ from biomass combustion

**DOI:** 10.1371/journal.pone.0180291

**Published:** 2017-07-03

**Authors:** Regina Dornhof, Christoph Maschowski, Anastasiya Osipova, Reto Gieré, Maximilian Seidl, Irmgard Merfort, Matjaz Humar

**Affiliations:** 1 Institute of Pharmaceutical Sciences, Pharmaceutical Biology and Biotechnology, Albert-Ludwigs University Freiburg, Freiburg, Germany; 2 Institute of Earth and Environmental Sciences, Albert-Ludwigs University Freiburg, Freiburg, Germany; 3 Department of Earth and Environmental Science and Center of Excellence in Environmental Toxicology, University of Pennsylvania, Philadelphia, Pennsylvania, United States of America; 4 Institute for Surgical Pathology, Faculty of Medicine, Albert-Ludwigs University Freiburg, Freiburg, Germany; 5 Center for Chronic Immunodeficiency (CCI), Faculty of Medicine, Albert-Ludwigs University Freiburg, Freiburg, Germany; 6 Spemann Graduate School of Biology and Medicine (SGBM), Albert-Ludwigs University Freiburg, Freiburg, Germany; Institute of Biochemistry and Biotechnology, TAIWAN

## Abstract

Fine particulate matter (PM_2.5_) can adversely affect human health. Emissions from residential energy sources have the largest impact on premature mortality globally, but their pathological and molecular implications on cellular physiology are still elusive. In the present study potential molecular consequences were investigated during long-term exposure of human bronchial epithelial BEAS-2B cells to PM_2.5_, collected from a biomass power plant. Initially, we observed that PM_2.5_ did not affect cellular survival or proliferation. However, it triggered an activation of the stress response p38 MAPK which, along with RhoA GTPase and HSP27, mediated morphological changes in BEAS-2B cells, including actin cytoskeletal rearrangements and paracellular gap formation. The p38 inhibitor SB203580 prevented phosphorylation of HSP27 and ameliorated morphological changes. During an intermediate phase of long-term exposure, PM_2.5_ triggered proliferative regression and activation of an adaptive stress response necessary to maintain energy homeostasis, including AMPK, repression of translational elongation, and autophagy. Finally, accumulation of intracellular PM_2.5_ promoted lysosomal destabilization and cell death, which was dependent on lysosomal hydrolases and p38 MAPK, but not on the inflammasome and pyroptosis. TEM images revealed formation of protrusions and cellular internalization of PM_2.5_, induction of autophagosomes, amphisomes, autophagosome-lysosomal fusion, multiple compartmental fusion, lysosomal burst, swollen mitochondria and finally necrosis. In consequence, persistent exposure to PM_2.5_ may impair epithelial barriers and reduce regenerative capacity. Hence, our results contribute to a better understanding of PM-associated lung and systemic diseases on the basis of molecular events.

## Introduction

Exposure to ambient particulate matter (PM) is associated with significant morbidity and mortality with approximately 7.2 million premature deaths due to outdoor and indoor air pollution [1, 2]. Particles less than 2.5 μm in diameter (PM_2.5_) are considered most harmful, as they penetrate deeply into the respiratory tract and adversely affect human health [3]. Emissions from residential energy sources used for cooking and heating globally have the largest impact on premature mortality connected e.g. to chronic obstructive pulmonary disease (COPD), acute lower respiratory illness, and ischaemic heart disease [1, 4, 5]. According to the WHO, 4.3 million people a year die from the exposure to household air pollution [6]. However, the involved molecular mechanisms remain largely unknown. As biomass combustion is increasingly used as a domestic or regenerative, CO_2_-neutral alternative energy source, adverse health effects of emissions from biomass combustion are an issue of growing concern.

Epithelial barriers of the respiratory system are directly exposed to inhaled atmospheric particles and probably display the earliest pathological changes. Recently it has been shown, that particles from cigarette smoke influence the architecture of the respiratory epithelium [7–9], which is controlled by multiple signaling pathways. RhoA, a small GTPase protein of the Rho family, is prevalent in regulating cell shape, polarity and locomotion via actin polymerization, actomyosin contractility, cell adhesion, and microtubule dynamics [10]. Upon acute cellular insults the p38 mitogen-activated protein kinase (p38 MAPK) mediates actin reorganization, stress fiber formation and cell migration, thus linking actin responses to external stimuli. Heat shock protein 27 (HSP27) is a direct target of p38 MAPK and has been suggested to have a homeostatic function by stabilizing actin microfilaments, accelerating their recovery after disruption and inhibiting apoptosis during cell stress [11, 12].

During stress, cells can actively suppress ATP-consuming metabolic processes and initiate ATP generating pathways to preserve the intracellular energy supply and to avert cellular damage [13, 14]. Here AMP-activated protein kinase (AMPK) plays a pivotal role by inhibiting protein synthesis at multiple points. Hence, this kinase initiates an inhibitory phosphorylation of eukaryotic elongation factor 2 (eEF2) [15–17], which is sufficient for translational inhibition [15, 18]. Repression of global protein synthesis prevents cell-cycle progression and depletion of energy metabolites, which then can be reallocated to vitality-preserving mechanisms and cellular repair [19–22]. Cell-cycle progression is also controlled by p38 MAPK in response to environmental stresses, e.g. by stabilization of the p21CIP1/WAF1 protein [23]. Energy homeostasis can also be sustained by autophagy [24]. Upon depletion of intracellular energy AMPK activates Unc-51-like kinase 1 (ULK1) [25]. Then, Atg1/ULK1 initiates the formation of the autophagosome, whereas Atg8/microtubule-associated protein light chain 3 (LC3) truncation and lipidation mediate autophagosome expansion [26]. However, impairment of the autophagolysosomal compartments may lead to the activation of the inflammasome [27, 28], and extensive autophagy has been associated with decreased cellular viability [29, 30].

Although the lung and airways are constantly exposed to ambient PM, an instantaneous impact on human health is rarely observed. Therefore, it can be assumed that cells utilize a cytoprotective adaptive stress response to protect themselves against adverse environmental conditions. To obtain a more detailed insight into how bronchial epithelial cells may counteract long-term exposure to PM, BEAS-2B cells were continuously exposed to PM_2.5_, emitted from a biomass combustion facility. We observed morphological changes and the induction of the typical adaptive stress response markers HSP27, p38, AMPK, and autophagy, but continuous exposure to PM_2.5_ resulted in senescence, autophagy and cell death. Images from transmission electron microscopy (TEM) gave insights into the cellular uptake of PM_2.5_ and supported the results on autophagy and necrosis. Altogether, our observations might explain PM-associated lung disorders by epithelial barrier dysfunction, reduced regenerative capacity, impaired autophagy, and extensive cell death.

## Materials and methods

### Preparation of fine particulate matter (PM_2.5_)

Bulk fly ash was collected from an electrostatic precipitator of a medium-scale biomass power plant with 1.7 MW nominal thermal output (Bürger Energie St. Peter eG, St. Peter, Schwarzwald, Germany), which exclusively combusts chips of soft wood (mainly spruce) derived from the local forests. The wood chips included debarked stem wood and branches with a minimum of 7 cm in diameter (merchantable wood) without leaves and twigs. Maximum combustion temperature was 910°C. The collected ash was subsequently size-fractionated by a cyclone with an aerodynamic cut-off diameter of 2.5 μm (Labor für Partikeltechnologie/Mechanische Verfahrenstechnik, Hochschule Konstanz, Technik, Wirtschaft und Gestaltung). The resultant size fraction (PM_2.5_) was then used for all biological assays.

### Cell culture and PM treatment

Immortalized bronchial epithelial cells (BEAS-2B; ATCC, Manassas, VA) were maintained in Dulbecco's Modified Eagle Medium / Ham’s F12 (GE Healthcare, Freiburg, Germany) containing 2 mM glutamine, 5 vol% fetal calf serum, 100 IU streptomycin and 100 IU penicillin (Life Technologies, Carlsbad, CA) at 37°C in a humidified incubator. BEAS-2B cells were plated at a density of 0.7 x 10^6^ cells per 75 cm^2^ cell culture flasks and were split twice a week using 0.25% trypsin/EDTA for detachment. After one day of recovery adherent cells were treated with PM_2.5_. Particles were immersed in BEAS-2B growth medium at a concentration of 200 μg/ml and sonicated for 20 min (SONOREX, BANDELIN electronic GmbH & Co. KG, Berlin, Germany) immediately before dilution to a final concentration of 100 μg/ml PM_2.5_. After addition of PM_2.5_, cells were maintained in the same medium until the next passage. For biological assays, cells were seeded in 6-well plates (immunoblotting), 12-well-plates (acridine orange) 24-well plates (actin cytoskeleton staining) or 96-well-plates (MTT assay) and exposed on the next day with 100 μg/ml PM_2.5_. Cells were harvested at times indicated for the respective experiment.

### Immunofluorescence staining of the actin cytoskeleton

BEAS-2B, seeded on cover slips (Roth, Karlsruhe, Germany), were fixed in 3.7 vol% formaldehyde and permeabilized with 0.1 vol% Triton X-100 for 10 min at room temperature. Then, cytoskeletal actin was stained with 0.2 U tetramethylrhodamine (TRITC) phalloidin (Life Technologies, Carlsbad, CA) for 40 min in the dark, before cells were mounted in ProLong^®^ Gold Antifade Mountant (Thermofisher Scientific, Waltham, MA) and examined with an Axiovert fluorescence microscope (Carl Zeiss Microscopy GmbH, Jena, Germany).

### Immunoblotting

Cells were lysed in 10 mM HEPES, pH 7.9, 350 mM NaCl, 1 mM MgCl_2_, 0.5 mM EDTA, 0.1 mM EGTA, 1% Nonidet P-40, 20% glycerol, 5 mM dithiotreitol, 2.5 mM phenylmethylsulfonyl fluoride, and 20 mg/ml aprotenin. Equal amounts of protein were resolved by SDS-PAGE, electrotransferred to polyvinylidene difluoride membranes (EMD Millipore, Billerica, MA) and probed with antibodies directed against p38 MAPK, phospho-p38 MAPK, phospho-HSP27(Ser82), p21 Waf1/CIP1, eEF2, phospho-eEF2(Thr56), phospho-AMPKα (Thr172) ULK1, phospho-ULK1(Ser555), LC3B, beclin-1, caspase-1, caspase-3, cleaved caspase-3, PARP, and α-tubulin according to the specifications of the manufacturer (Cell Signaling Technology, Danvers, MA). The antibody detecting GAP-DH was from EMD Millipore, the antibody detecting RhoA was from Santa Cruz Biotechnology (Santa Cruz Biotechnology, Santa Cruz, CA). Specific protein bands were visualized using horseradish-peroxidase conjugated anti-rabbit IgGs and enhanced chemiluminescence reagents (GE Healthcare, München, Germany).

### RhoA activity assay

Rho GTPase pulldown experiments were performed as described previously [31]. Briefly, BEAS-2B cells were harvested and lysed in ice-cold extraction buffer, containing 10% glycerol, 50 mM Tris pH 7.4, 100 mM NaCl, 1% NP-40, 2 mM MgCl_2_, and 1 mM PMSF. After centrifugation at 15,000 g, the supernatant was mixed 1: 1 (*v/v*) with glutathione-sepharose beads bound to GST-Rhotekin (kindly provided by Prof. Dr. G. Schmidt, Institute for Experimental and Clinical Pharmacology and Toxicology, University of Freiburg) and rotated on a wheel at 4°C for 1 h. Beads bound to the active form of RhoA were washed twice with extraction buffer and boiled in SDS sample buffer. Active, precipitated RhoA was visualized by immunoblotting. Crude cellular lysates were used to determine the total amount of RhoA proteins in each sample.

### Cell-cycle analysis

BEAS-2B were fixed in 70 vol% ice cold ethanol overnight and subsequently suspended in phosphate buffered saline, containing 0.1 mg/ml RNase and 0.25 mg/ml propidium iodide. Cell cycle distribution was examined by measuring the DNA content by a FACS Calibur (BD Biosciences, Heidelberg, Germany). The percentage of cells in the G0/G1, S and G2/M phases, as well as the percentage of apoptotic cells in the ‘‘sub-G1” peak, were quantified using the BD Cell Quest^™^ Pro software (BD Biosciences).

### Cellular viability assay

Cell viability of BEAS-2B cells was determined by the MTT [3-(4,5-dimethylthiazol-2-yl)-2,5-diphenyltetrazolium bromide] assay as described previously [32]. Briefly, cells were seeded at a density of 2500 cells per well of a 96-well flat-bottomed cell culture plate and maintained at 37°C, 5% CO_2_ overnight. The inhibitors SB203580 (10 μM; EMD Millipore), E-64-D (10 μM; Enzo Life Sciences, Lausen Switzerland), Pepstatin (5 μM; Roche Diagnostics, Mannheim, Germany), Dorsomorphin (1 μM; Sigma-Aldrich, Munich, Germany) or Z-WEHD-FMK (10 μM; R&D Systems, Wiesbaden-Nordenstadt, Germany) were added 1 h prior to re-exposition of BEAS-2B cells with 100 μg/ml of PM_2.5_. After 72 h, 0.5 mg/ml MTT was added for 2 h before formazan crystals were dissolved in 100% DMSO. Absorbance at 595 nm was determined with a BIO-RAD iMark^™^ Microplate Reader (BIO-RAD, Hercules, CA). Cell viability was displayed as the percentage of untreated control cells.

### Analysis of lysosomal membrane permeabilisation

Cells were incubated with 1 μg/ml acridine orange (Sigma-Aldrich) for 15 min at 37°C in a CO_2_ incubator. Cells were washed with PBS and incubated with 1 μg/ml acridine orange in FACS buffer (PBS + 3% fetal calf serum) for 15 min at 37°C in a CO_2_ incubator. Subsequently, cells were washed twice with PBS, trypsinized, and resuspended in FACS buffer. Cells were analyzed by flow cytometry. At least 10,000 events were analyzed. Lysosomal permeabilisation was evaluated by measuring the loss of emission at 600–650 nm using flow cytometry (FACS Calibur, BD Biosciences).

### Detection of IL-1β

Release of mature IL-1β was determined in cell culture supernatants using the human IL-1 beta/IL-1F2 Quantikine sandwich ELISA kit according to the description of the manufacturer (R&D Systems GmbH). Transcription of the IL-1β gene was quantified by qRT-PCR. Briefly, total RNA was isolated from cells using the RNeasy^®^ Plus Mini Kit and converted to single strand cDNA using the QuantiTect^®^ Reverse Transcription Kit according to the instructions of the manufacturer (Qiagen, Hilden, Germany). The cDNA was amplified in a Light Cycler^®^ 480 (Roche Diagnostics, Basel, Switzerland) using 2 x conc. LightCycler^®^ 480 Probes Master, 50 nM primers and 100 nM probe for the 18S rRNA reference gene (fwd: 5’-CGGCTACCACATCCAAGG-3’, rev: 5’-CGGGTCGGGAGTGGGT-3’, probe: 5’-[HEX]-TTGCGCGCCTGCTGCCT-[TAM]-3’) or 300 nM primers and 200 nM probe for the human IL-1β gene (fwd: 5’-GTACGATCACTGAACTGC-3’, rev: 5’-GTGGAGAGCTTTCAGTTC-3, probe: 5’-[6-FAM]-ATGGACCAGACATCACCAAGC-[TAM]-3’). For relative quantification ΔC_T_ values of PM_2.5_-treated cells were referred to untreated control cells resulting in a ΔΔC_T_ value. The fold increase was calculated as 2^-ΔΔCT^.

### TEM analysis

PM_2.5_-exposed cells were seeded into 6-well cell culture inserts (200.000 cells per insert) (pore size 3 μm; Corning, New York, USA) overnight (for culture conditions see Material and methods). Subsequently, the cell-bearing insert membrane was carefully detached from the insert. Samples of ~1 x 5mm were fixed in 3% glutaraldehyde, Dulbecco’s PBS, and 0.5% osmium tetroxide / 0.025 M potassium hexacyanoferrate(III) solution, followed by incubation in uranyl acetate / 70% ethanol overnight. Samples were then dehydrated in ascending ethanol solutions starting from 80% ethanol and embedded in epoxy resin (EPON) blocks. Subsequently, blocks were cut in smaller blocks of ~1mm diameter. Ultrathin sections (80nm) were taken with a diamond blade from EPON blocks (Reichert ULTRACUT, Leica, Wetzlar, Germany), mounted on a 200 mesh hexagonal platinum grid and further contrasted in a 0.2% lead citrate / 0.1 M sodium hydroxide solution for 20 min. Photos were taken with the MORADA camera (Olympus Soft Imaging System, Münster, Germany) using a transmission electron microscope (FEI Thermo Fisher Scientific, Munich, Germany) with magnifications as depicted.

### Statistics

Values are shown as mean ± standard deviation (SD) for the indicated number of independent experiments. Statistical analysis was performed by the GraphPad PRISM^®^ 5 software (GraphPad Software, San Diego, CA) using 1-way or 2-way ANOVA followed by the Bonferroni’s post-hoc test or the student’s t-test. *, p < 0.05; **, p < 0.01; ***, p < 0.001 were considered as significant.

## Results

### Isolation and characterization of PM_2.5_ from biomass combustion

PM_2.5_ was investigated by scanning electron microscopy (SEM), X-ray diffraction (XRD) and Rietveld refinement to characterize the physical and chemical properties of the individual particles and to determine their mineralogical identity. Particles displayed various shapes and sizes with different chemical compositions, as observed by SEM (S1 File). PM_2.5_ mainly consisted of crystalline arcanite (K_2_SO_4_, 47 wt%) and amorphous components (30 wt%), including typical fly ash spheres, and some organic materials. In addition to arcanite, the crystalline fraction also included other sulfates, as well as sylvite (KCl), carbonates, oxides, hydroxides, and silicates (S1 Fig).

### Long-term exposure to PM_2.5_ affects cell morphology, actin organization and induces the stress-responsive p38 MAPK

**Fig 1:**
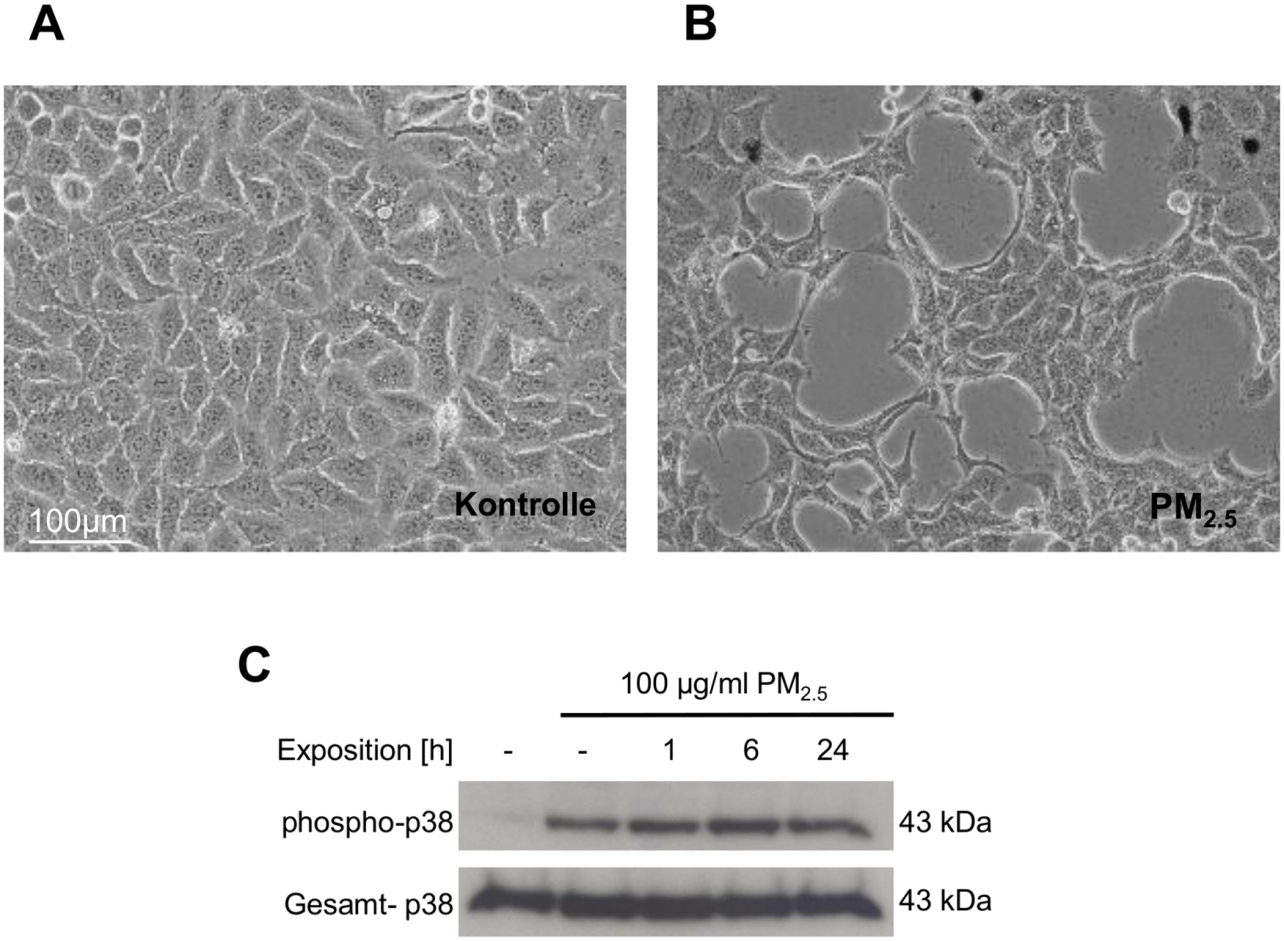
PM_2.5_-induced morphological changes in shape and paracellular gap formation and activation of p38 MAPK. Photomicrographs at 200x magnification from (**A**) untreated BEAS-2B cells or (**B**) cells exposed to 100 μg/ml of PM_2.5_ for 5 weeks. (**C**) Phosphorylation of p38 MAPK at Thr180/Tyr182 upon exposure to PM_2.5_. BEAS-2B cells were left untreated (lane 1), exposed to 100 μg/ml of PM_2.5_ for 5 weeks and then particles were removed for 24 h (lane 2), or cells were re-exposed to PM_2.5_ for 1 to 24 h (lanes 3–5). The immunoblots were normalized using an antibody, detecting total p38 MAPK. Results are shown from 5 (**A**, **B**) and 3 (**C**) different experiments.

The PM_2.5_ fraction was used for continuous exposure of human bronchial epithelial BEAS-2B cells to simulate recurrent inhalation of xenobiotic airborne particles. Within the first 5 weeks, PM_2.5_-exposed BEAS-2B cells did not display reduced proliferation or morphological signs of cytotoxicity, but acquired an elongated, spindle-like appearance, whereas untreated control cells maintained their original, epithelial-like cuboidal shape (Fig 1A and 1B). BEAS-2B, grown in the presence of PM_2.5_ were additionally found to form narrow structures of tightly clustered cells and large paracellular gaps. These gaps were already visible within 3 weeks, but more pronounced after 5 weeks.

**Fig 2:**
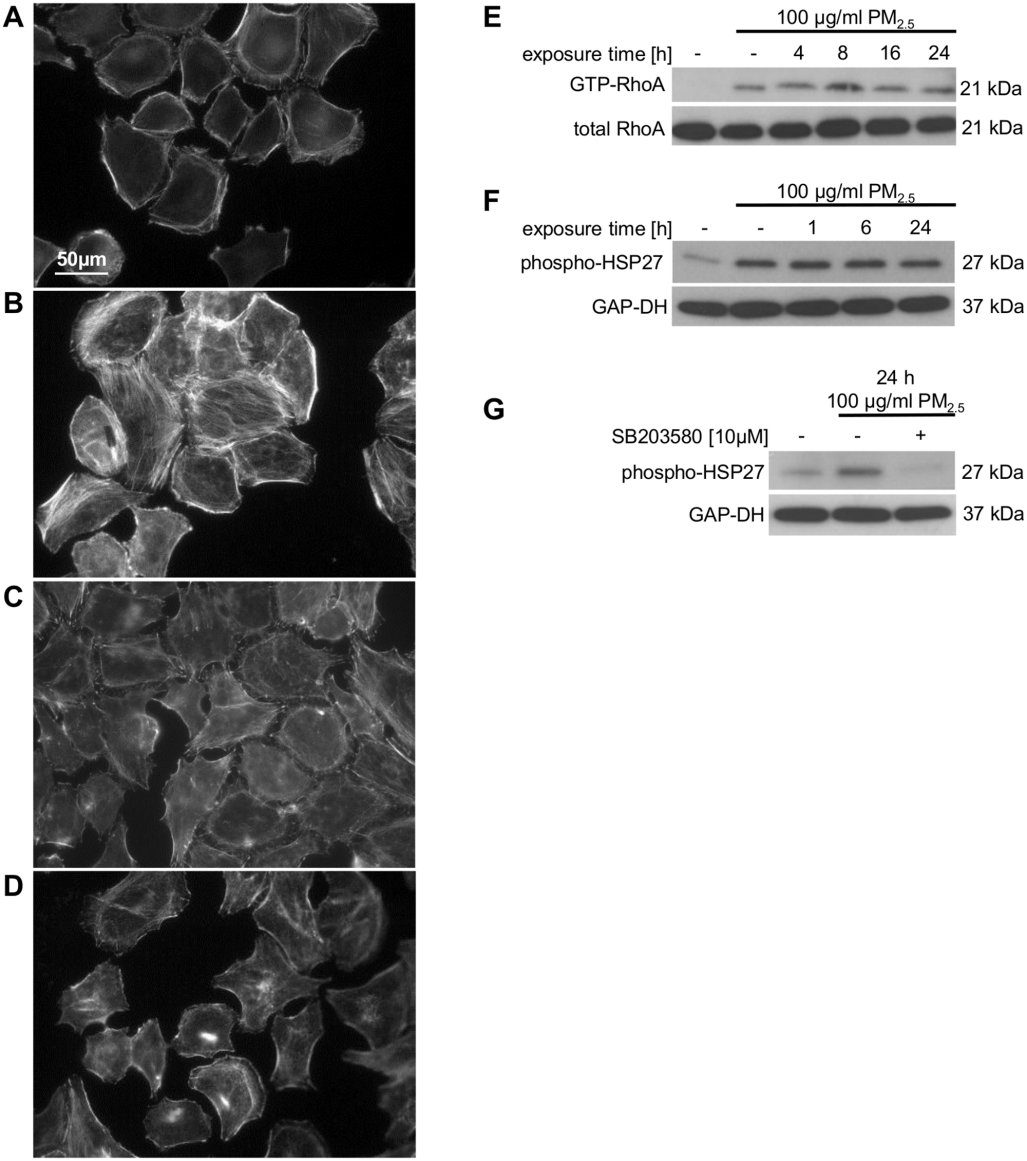
Effect of PM_2.5_ on the actin cytoskeleton, p38 MAPK and HSP27. Microscopy of BEAS-2B cells after staining with 0.2 U/ml TRITC phalloidin for 40 min (n = 2). Cells not treated with PM_2.5_ (**A**, **C**), cells re-exposed to 100 μg/ml of PM_2.5_ for 8 h after long-term culture with PM_2.5_ for 5 weeks (**B**, **D**). The p38 MAPK inhibitor SB203580 (10 μM) was added 1 h prior to re-exposure to PM_2.5_ (**C**, **D**). Effect of PM_2.5_ on RhoA activity and HSP27 phosphorylation (**E–G**). BEAS-2B cells were left untreated or were exposed to 100 μg/ml PM_2.5_ for 5 weeks before removal of particles for 24 h or re-exposure to 100 μg/ml of PM_2.5_ for 1–24 h. Immunoblots show precipitation of active RhoA with GST-Rothekin from total cellular lysates (upper panel) and total amount of RhoA in cellular lysates (lower panel), (**E**; n = 3); phosphorylation of HSP27 at Ser82, (**F**; n = 3), addition of 10 μM of the p38 MAPK inhibitor SB203580 1 h prior to re-exposure to PM_2.5_ (**G**; n = 2). GAP-DH was used as a loading control.

The phenotypic morphological changes pointed to a stress response and a rearrangement of the actin cytoskeleton. Therefore, we analyzed the effect of PM_2.5_ on the stress-induced p38 MAPK by immunoblotting and on the intracellular actin cytoskeleton by Texas Red phalloidin staining. Continuous exposure to PM_2.5_ was accompanied by a prolonged phosphorylation and therefore activation of p38 MAPK, which was still present after removing the particles for 24 h (Fig 1C, lane 2). Moreover, PM_2.5_ affected the molecular structure of the intracellular actin cytoskeleton by inducing the formation of stress fiber bundles, which disappeared in the presence of the p38 MAPK inhibitor SB203580, indicating a prominent role of this stress-induced kinase in cytoskeletal reorganization (Fig 2A–2D).

### Long-term exposure to PM_2.5_ induces RhoA and phosphorylation of HSP27

The small GTPases of the Rho family are master regulators of dynamic cytoskeletal actin structures and associated with stress fiber formation [10]. Pull-down experiments using the Rho-binding domain of Rothekin demonstrated the precipitation of GTP-bound (active) RhoA only in lysates of PM_2.5_-exposed BEAS-2B cells (Fig 2E). RhoA GTPases remained active even after removal of particles for at least 24 h (Fig 2E, lane 2) and showed a response to PM_2.5_ that is similar to the activation of the stress-induced p38 MAPK (Fig 1C).

HSP27 is known as a substrate of the p38 MAPK pathway and is activated by phosphorylation at serine 82 upon stressful conditions to maintain cellular homeostasis by preservation of the integrity of actin and the intermediate filaments [11, 12]. Immunoblot analysis revealed, that similar to RhoA or p38 MAPK, phosphorylation of HSP27 at serine 82 is increased in PM_2.5_-exposed cells and remained even after removal of the particles (Fig 2F, lane 2). Treatment with the p38 MAPK inhibitor SB203580 prevented the observed phosphorylation of HSP27 in PM_2.5_-exposed cells (Fig 2G, lane 3). Both, the restoration in cell morphology and the blockade of the HSP27-Ser82 phosphorylation in SB203580-treated, PM_2.5_-exposed BEAS-2B cells implicate a functional role of HSP27 in the p38 MAPK-dependent rearrangement of the actin cytoskeleton.

### Long-term exposure to PM_2.5_ affects cell-cycle progression

**Fig 3:**
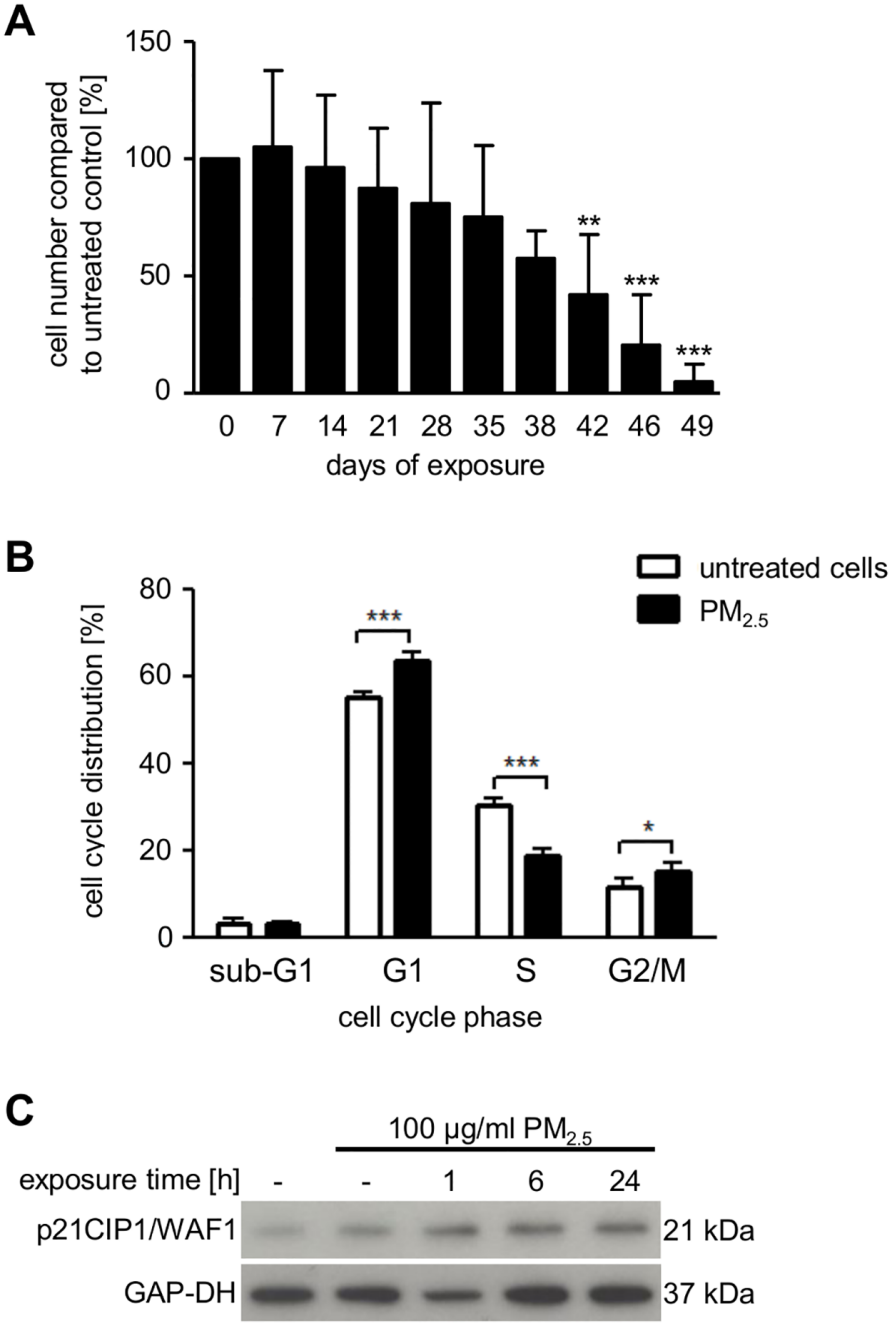
Effect of PM_2.5_ on cell numbers and cell cycle. (**A**) BEAS-2B cells counted by a trypan blue exclusion assay using a Neubauer counting chamber every three days after passage and exposure to PM_2.5_. Statistically significant differences represent the mean ± SD, n = 5 (1-way ANOVA, **, *p* < 0.01; ***, *p* < 0.001) (**B**) Cell cycle distribution analyzed by flow cytometry after 3–4 weeks of exposure to PM_2.5_ and 72 h re-exposure to PM_2.5_. Results are displayed as percentage of PM_2.5_-exposed cells compared to untreated control cells. Statistically significant differences represent the mean ± SD, n = 4 (2-way ANOVA followed by the Bonferroni’s post-hoc test, *, *p* < 0.05; ***, *p* < 0.001). (**C**) Accumulation of p21CIP1/WAF1, analyzed by immunoblotting. Untreated cells (lane 1), exposed to 100 μg/ml PM_2.5_ for 3–4 weeks before removal of particles for 24 h (lane 2), or re-exposed to 100 μg/ml of PM_2.5_ for 1–24 h (lanes 3–5). GAP-DH served as a loading control (n = 3).

After 5 weeks of continuous exposure to PM_2.5_ (intermediate phase), we observed a decrease in cell number, and finally, nearly all of the cells were lost in the presence of PM_2.5_ (Fig 3A). Cell-cycle analysis performed after 5–7 weeks revealed a significant G1 and G2/M phase arrest and reduced cell numbers in the S phase (data not shown). This was already the same after 3–4 weeks (Fig 3B), suggesting that long-term exposure to PM_2.5_ impairs cell-cycle progression by preventing the transition at various cell-cycle checkpoints. Decreased incorporation of 5-bromo-2-deoxyuridine into replicating DNA of PM_2.5_-exposed cells confirmed reduced proliferation (S2 Fig). The absence of a significant increase in cell numbers during the sub-G1 phase of PM_2.5_-treated BEAS-2B cells indicated that reduced cell numbers were not attributed to cell death (Fig 3B).

The p38 MAPK can regulate several cell-cycle phases in response to various stresses, e.g. by stabilization of the CDK inhibitor p21CIP1/WAF1 [23]. Consistently, continuous exposure to PM_2.5_ increased expression of p21CIP1/WAF1 (Fig 3C), which might explain both G1/S phase and G2/M phase restriction.

### Long-term exposure to PM_2.5_ activates AMPK

**Fig 4:**
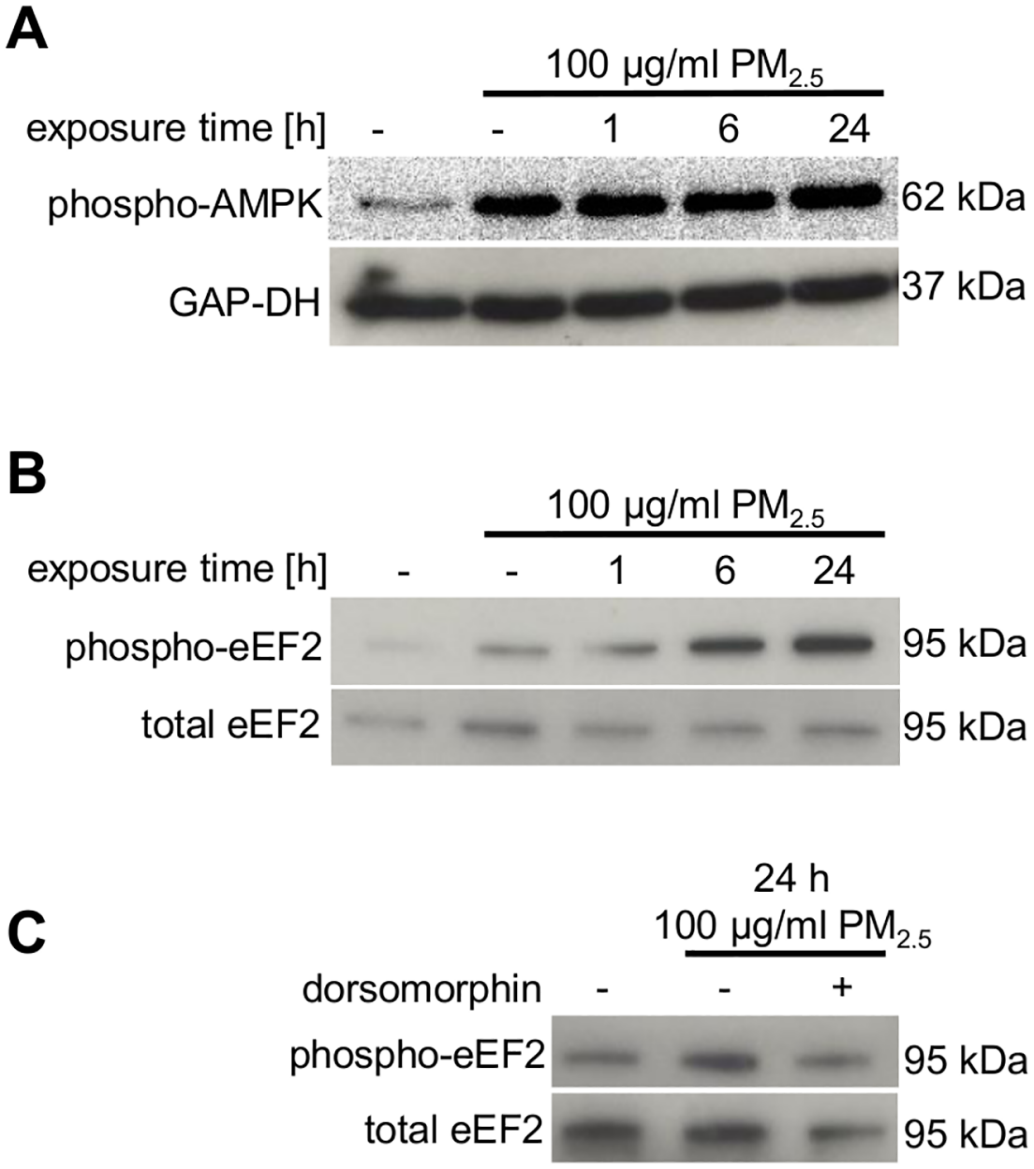
Effect of PM_2.5_ on AMPK and eEF2 in BEAS-2B cells. Cells were untreated or exposed to 100 μg/ml PM_2.5_ for 5–7 weeks before removal of particles for 24 h (lane 2) or re-exposure to 100 μg/ml PM_2.5_ for 1–24 h (lanes 3–5). (**A)** Immunoblotting of the activating phosphorylation of AMPK at Thr172 (n = 3) and (**B**, **C**) the inhibitory phosphorylation of eEF2 at Thr56. The AMPK inhibitor dorsomorphin (10 μM) was added 1 h prior to re-exposure to PM_2.5_. (n = 2) Detection of GAP-DH or total eEF2 was used as loading controls.

Since cell-cycle progression depends on availability of cellular energy, we determined the consequences of long-term exposure to PM_2.5_ on AMPK which is a crucial regulator of energy homeostasis [20]. We observed a prolonged phosphorylation and therefore activation of AMPK (Fig 4A) which was still present after removing the particles for 24 h (Fig 4A, lane 2). Moreover, eEF2, a substrate of AMPK-dependent eEF2 kinase, was also phosphorylated and thereby inactivated (Fig 4B). This phosphorylation was reduced in the presence of the selective AMPK-inhibitor dorsomorphin, indicating that AMPK is involved in the inactivation of eEF2 (Fig 4C, lane 3). When intracellular energy supply is limited, activation of AMPK and inactivation of eEF2 represent pivotal stress responses, which redirect limited energy sources to pathways necessary for cellular survival [20]. Phosphorylation of eEF2 at threonine 56 is associated with inhibition of global protein synthesis, which might contribute to the observed arrest at multiple cell-cycle checkpoints, as observed in Fig 3B [21].

### Long-term exposure to PM_2.5_ promotes autophagy and cell death

**Fig 5:**
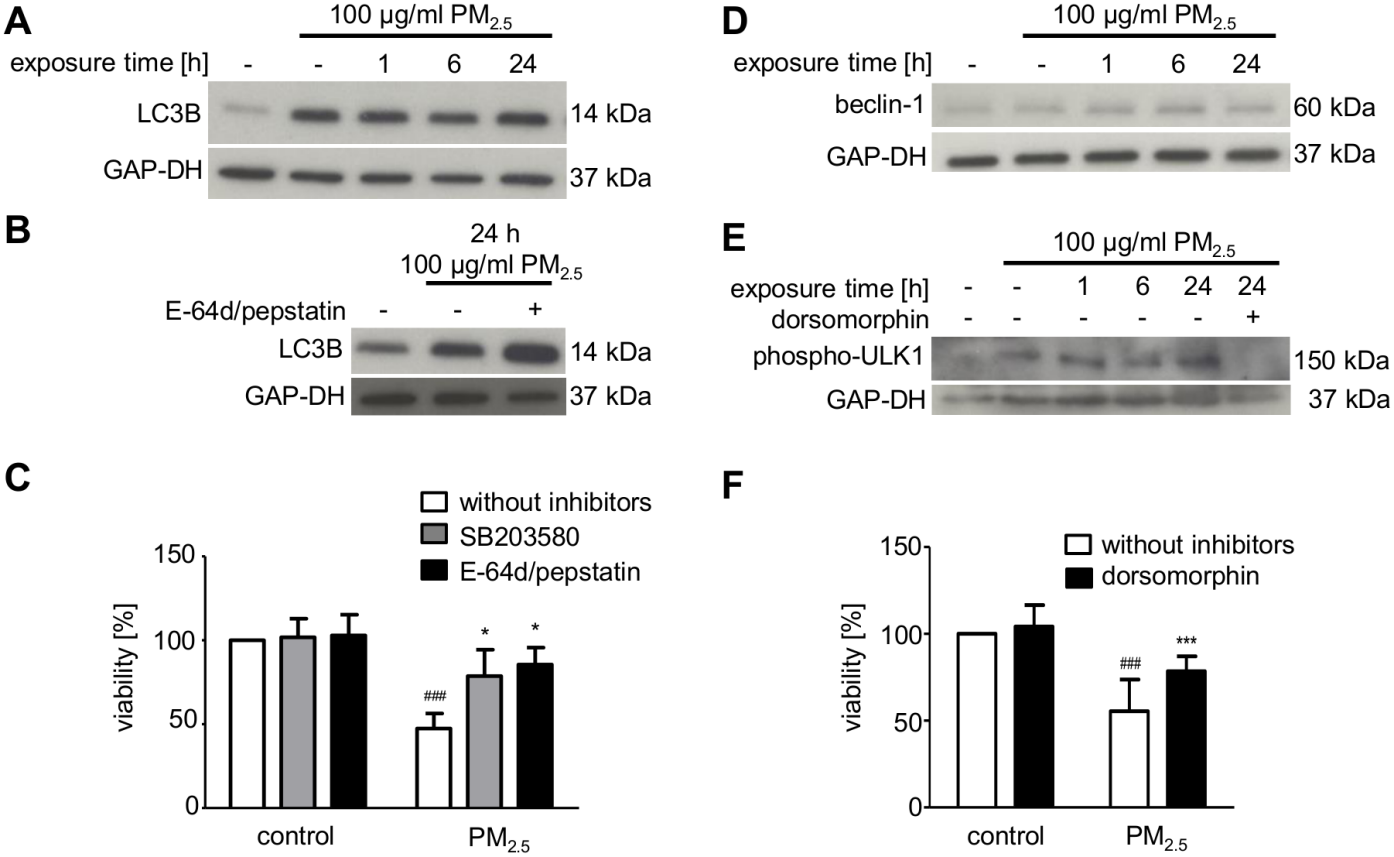
Effect of PM_2.5_ on autophagy and cell viability. Autophagy was studied by immunodetection of, truncated LC3B (**A**, **B**), beclin-1 up-regulation (**D**), or ULK-1 phosphorylation at Ser555 (**E**) in whole cell lysates of BEAS-2B cells, left untreated or exposed to 100 μg/ml PM_2.5_ for 5–7 weeks before removal of particles for 24 h or re-exposure to 100 μg/ml PM_2.5_ for 1–24 h. Autophagy was prevented by 10 μM E-64d plus 5 μM pepstatin, added 1 h before re-exposure to PM_2.5_ (**B**). The AMPK inhibitor dorsomorphin (10 μM) was added 1 h prior to re-exposure to PM_2.5_ (**E**). Detection of GAP-DH served as a loading control. (**A**, **B**, **D**, **E**) MTT cell viability assay was performed one week before complete cell death of BEAS-2B cells after persistent treatment with 100 μg/ml PM_2.5_ and re-exposure for 72 h. SB203580 (10 μM), E-64d (10 μM) plus pepstatin (5 μM) (**C**) or dorsomorphin (1 μM) (**F**) were added 1 h prior to addition of PM_2.5_. Results, displayed as percentage of MTT conversion of PM_2.5_-exposed cells compared to untreated control cells. Statistically significant differences within groups are shown for BEAS-2B cells, left untreated or exposed to 100 μg/ml PM_2.5_ (^###^, *p* < 0.001) and PM_2.5_-exposed cells vs. PM_2.5_-exposed cells in the presence of 10 μM SB203580 (*, *p* < 0.05), 10 μM E-64d plus 5 μM pepstatin (**, *p* < 0.01) or 1 μM dorsomorphin (***, *p* < 0.001). **A**–**D**: n = 3, **E**: n = 2, **F**: n = 3; statistical analysis by 2-way ANOVA followed by the Bonferroni’s post-hoc test, respectively.

Activation of AMPK and inactivation of eEF2 point to a metabolic stress response, which can be associated with autophagy [33]. Moreover, particles are reported to induce autophagy as an attempt to degrade foreign material [34]. Accordingly, treatment of BEAS-2B with PM_2.5_ resulted in a marked increase in truncated LC3B (Fig 5A), which is required for the formation of autophagosomal vacuoles [35]. E-64d and pepstatin, which inhibit lysosomal cathepsins, further increased the expression of LC3B, indicating that accumulation of autophagosomes was due to increased formation rather than impaired degradation (Fig 5B). The consequences of autophagy induction were further analyzed by using the MTT cell viability assay. PM_2.5_ significantly diminished cell viability of continuously exposed BEAS-2B cells (Fig 5C). Cell viability was increased, when lysosomal cathepsins and autophagy were inhibited by the addition of E-64d and pepstatin (Fig 5C). Accordingly, autophagy induced by PM_2.5_ promotes cell damage, consistent with the dramatic loss in cell numbers displayed in Fig 3A.

As inhibition of the stress-responsive p38 MAPK ameliorated morphological changes by PM_2.5_ (Fig 2D), we investigated whether or not p38 MAPK is also involved in the impaired viability of PM_2.5_-treated BEAS-2B cells. Inhibition of p38 MAPK by SB203580 significantly increased the viability of PM_2.5_-treated cells. The increase was comparable in magnitude when autophagy and lysosomal hydrolases were inhibited (Fig 5C), which suggests that both processes may be related. p38 MAPK can induce autophagy via p53-mediated beclin-1 synthesis [36], but no marked increase in beclin-1 protein levels could be observed in cells that were continuously exposed to PM_2.5_ for 5–7 weeks (Fig 5D). However, we observed phosphorylation of ULK1 in BEAS-2B cells exposed to PM_2.5_ (Fig 5E). ULK1 together with Atg1 is essential for autophagy induction and regulation, and its activating phosphorylation at serine 555 is mediated by AMPK, which is induced by PM_2.5_ (Fig 4A) [25]. Consequently, the AMPK-inhibitor dorsomorphin prevented phosphorylation and thus activation of ULK1 at serine 555 (Fig 5E) and increased viability (Fig 5F).

**Fig 6:**
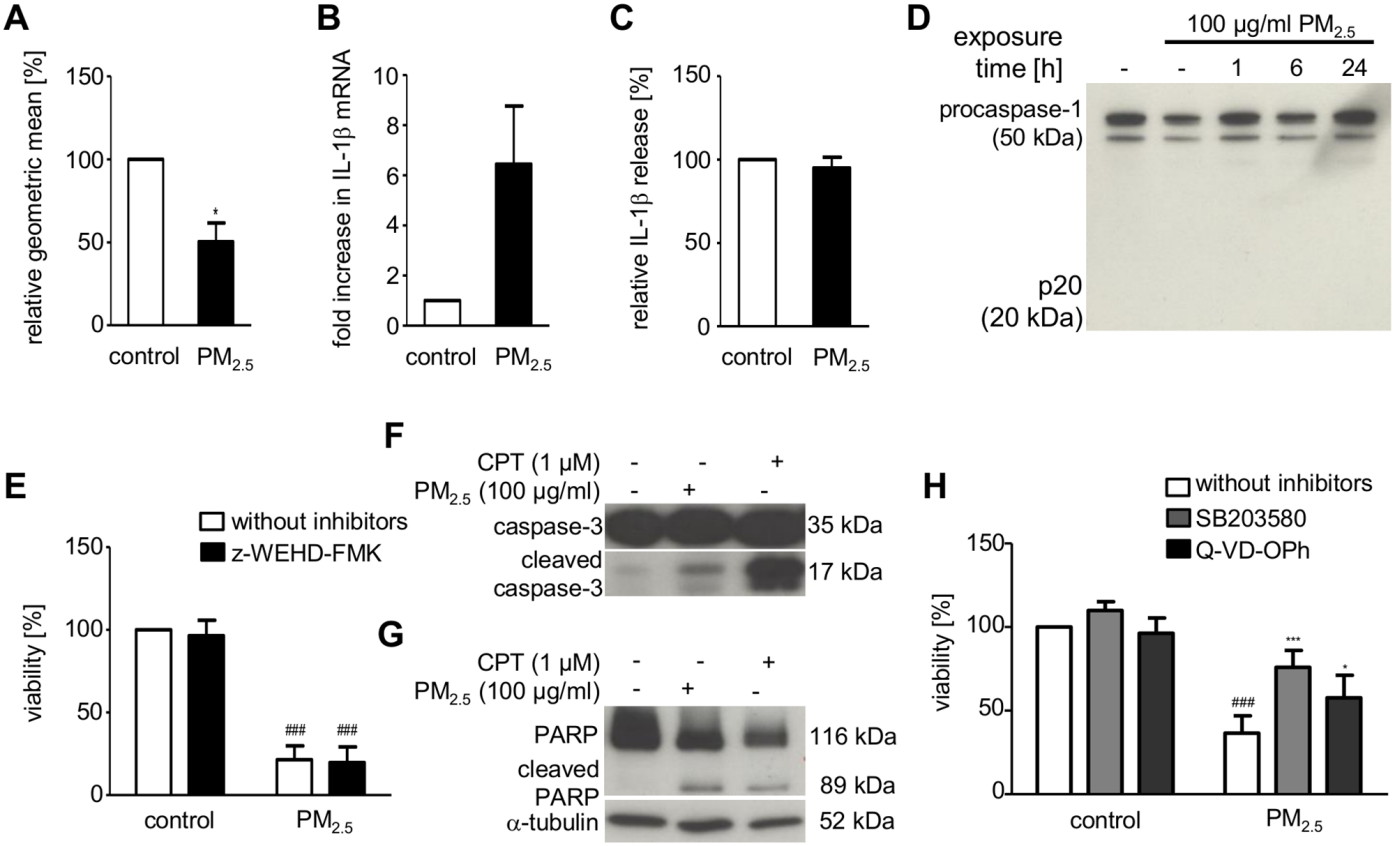
Effect of PM_2.5_ on lysosomal integrity, inflammasome activation, and apoptosis in BEAS-2B cells. Cells were left untreated or exposed to 100 μg/ml PM_2.5_ one week before complete cell death. (**A**) Acridine orange staining and flow cytometry to determine lysosomal destabilization 72 h after re-exposure to PM_2.5_. Fluorescence intensity, displayed as the geometric mean of PM exposed BEAS-2B cells vs. untreated control cells (student’s t-test, *, *p* < 0.05; n = 3). (**B**) qRT-PCR for IL1β gene expression and (**C**) IL1β ELISA to quantify extracellular release of mature IL-1β 48 h after re-exposure to PM_2.5_ (student’s t-test, n = 3). (**D**) Immunodetection of procaspase-1 and its cleaved, activated fragment (p20) in whole cell lysates 0–24 h after re-exposure to PM_2.5_. (**E**) MTT cellular viability assay using the caspase-1 inhibitor z-WEHD-FMK (10 μM), added 1 h prior to re-exposure to PM_2.5_ for 72 h. Results show percentage of MTT conversion compared to untreated control cells (2-way ANOVA followed by the Bonferroni’s post-hoc test, ^###^, *p* < 0.001; n = 3). (**F**) Immunodetection of procaspase-3 processing to its active, cleaved form and (**G**) cleavage of the cellular caspase-3 substrate PARP in whole cellular lysates, prepared 24 h after re-exposure to PM_2.5_ (n = 3). Detection of α-tubulin served as a loading control. (**H**) MTT cellular viability assay using the p38 inhibitor SB203580 (10 μM) or the caspase-3/7 inhibitor Q-VD-OPh (10 μM), added 1 h prior to re-exposure to PM_2.5_ for 72 h. Statistically significant differences within groups are shown for BEAS-2B cells, left untreated or exposed to 100 μg/ml PM_2.5_ (2-way ANOVA followed by the Bonferroni’s post-hoc test, ^###^, *p* < 0.001) and PM_2.5_-exposed cells vs. PM_2.5_-exposed cells in the presence of SB203580 (2-way ANOVA followed by the Bonferroni’s post-hoc test, ***, *p* < 0.05) or Q-VD-OPh (2-way ANOVA followed by the Bonferroni’s post-hoc test, *, *p* < 0.001); n = 3.

Intracellular accumulation of indigestible inorganic particles might lead to destabilization, or even permeabilization of PM-engulfing intracellular compartments, release of hydrolytic enzymes, and ultimately cell death [27]. Indeed, inhibition of lysosomal enzymes by E-64d and pepstatin improved viability of PM_2.5_-exposed BEAS-2B cells, indicating that lysosomal enzymes, such as cathepsins, might be involved in the progression of PM_2.5_-induced cellular damage (Fig 5C). Therefore, we investigated the effect of continuous PM_2.5_ exposure on lysosome membrane integrity by lysosomal staining using acridine orange followed by flow cytometry for quantification. Loss of acridine fluorescence intensity was observed, indicating a reduced number of lysosomes, e.g. due to lysosomal rupture (Fig 6A).

Cytosolic activity of leaked lysosomal enzymes has been proposed as a mechanism for inflammasome activation and cell death by pyroptosis [27, 28]. Therefore, we analyzed whether exposure to PM_2.5_ induced characteristic features of the inflammasome, such as activation of caspase-1 or release of IL-1β. Although we observed an increase in IL-1β gene transcription (Fig 6B), we could neither detect caspase-1 cleavage (Fig 6D) nor release of mature IL-1β into the supernatants of BEAS-2B cells that were continuously exposed to PM_2.5_ (Fig 6C). Accordingly, PM_2.5_-induced cell damage could not be ameliorated by z-WEHD-FMK, a caspase-1 inhibitor (Fig 6E). However, loss of viability by PM_2.5_ was accompanied by caspase-3 activation and PARP cleavage, two specific markers of cell death mediated by apoptosis (Fig 6F and 6G). Q-VD-OPh, a pan-caspase inhibitor that protects cells from caspase-dependent apoptosis, partially restored viability (Fig 6H). This indicates that apoptosis is only partially contributing to PM-mediated cell death and that pyroptosis is not involved.

### TEM visualized internalization of PM_2.5_, formation of amphisomes and complex fusion products, their rupture and necrosis

**Fig 7:**
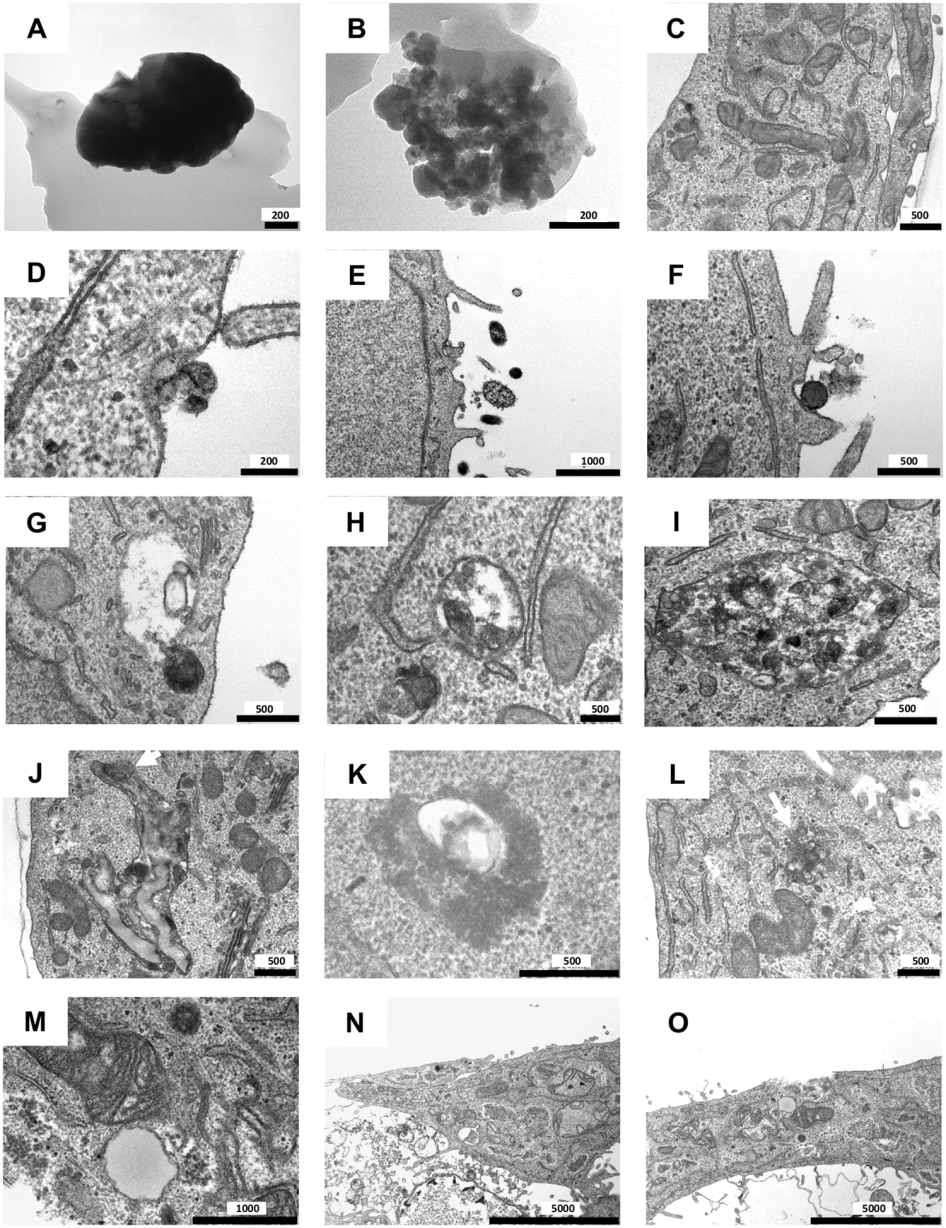
Representative TEM images of BEAS-2B cells after short-term exposure (D–I) or long-term exposure (J–O) to PM_2.5_ (100 μg/ml). (**A**, **B**) PM_2.5_ and (**C**) BEAS-2B cells without PM_2.5_ as controls. (**D–F**) Internalization mechanism of PM_2.5_. (**G**) Fusion of a PM_2.5_ containing membrane-bound vesicle with an autophagosome (double membrane vesicle). (**H**) Vesicle (amphisome) after fusion of a membrane-bound vesicle with PM_2.5_ and an autophagosome. (**I**) Complex fusion product with PM_2.5_. (**J**) Large fusion products containing PM_2.5_. The white arrow indicates a ruptured membrane. (**K**) Amphisome/fusion product with PM_2.5_, released into the cytosol. (**L**) White arrow indicates released PM_2.5_ from the fusion products in the cytosol. (**M**) Swollen mitochondria. (**N**, **O**) Necrotic BEAS-2B cells. Scale bars are indicated in nm.

TEM was utilized to investigate the cellular uptake of PM_2.5_, which appeared either as individual particles of electron-dense crystalline material or as aggregates (Fig 7A and 7B). Within days, PM_2.5_ was internalized by BEAS-2B cells accompanied either by cell-membrane invagination (Fig 7D) or, more commonly, by formation of protrusions (Fig 7E and 7F). In the cytoplasma, PM_2.5_ was localized within membrane-bound vesicles, which fused with autophagosomes to form amphisomes (exemplarily depicted by Fig 7G–7I). Besides amphisomes (Fig 7H), complex fusion products with PM_2.5_ could be observed, which probably arose from further fusions with amphisomes and lysosomes (Fig 7I). The content of these fusion products appeared more fragmented than the amphisomes, indicating degradation. Interestingly, complex fusion vesicles were also detected at the surface of the cell membrane (S3 Fig), pointing to extracellular release to remove indigestible particles [37]. Ongoing exposure to PM_2.5_ appeared to increase the number of membrane-bound particle vesicles, amphisomes and complex fusion products (S4B–S4E Fig).

Particles that fail lysosomal degradation may result in multiple compartmental fusion and formation of larger vacuoles, which include multiple particles and remnants of cellular organelles [37]. Accordingly, we also observed these larger vacuoles (Fig 7J), in which particles have been sequestered after long-term PM_2.5_-exposure. Additionally, overloading of the complex fusion products with PM_2.5_ led to rupture of the vacuole membranes and release of PM_2.5_ into the cytosol (Fig 7J–7L). Consequently, the number of lysosomes will decrease, as shown by reduced acridine orange staining in Fig 6A. Compared to control cells (Fig 7C), the morphological structure of the mitochondria progressively changed during long-term exposure to PM_2.5_. During ongoing exposure to PM_2.5_, mitochondria first developed swollen features (S4B Fig), increasing deformation and finally rupture of the cristae (Fig 7M). Functional impairment of the mitochondria may result in intracellular energy depletion and consequently activation of AMPK [16], which we observed in Fig 4A. Furthermore, lysosomal rupture and dysfunctional mitochondria may cause cells to undergo necrotic cell death [37, 38], which we were able to demonstrate (Fig 7N and 7O).

## Discussion

To explain the observed adverse health effects of emissions from biomass combustion, we determined the molecular consequences of long-term exposure of human bronchial epithelial BEAS-2B cells to PM_2.5_ from biomass combustion.

Initially, prolonged exposure to PM_2.5_ resulted in extensive morphological changes and in activation of the stress-responsive proteins p38 MAPK, RhoA and HSP27. The three proteins are known to regulate cytoskeletal structure and organization, indicating their functional participation in PM-mediated cytoskeletal re-architecture [10, 12, 39, 40]. Activation of RhoA may also be connected to the formation of protrusions, an actin-dependent process, which needs RhoA [41]. These protrusions participate in the internalization process of PM_2.5_ which we could clearly observe by TEM, and which are also discussed as a natural defense mechanism of cells against unwanted objects [42]. The central role of p38 MAPK in morphological changes was confirmed by a specific p38 MAPK inhibitor, which reversed the cytoskeleton changes. Despite extensive morphological changes cellular proliferation and viability were not significantly affected. The missing cytotoxicity contrasts various reports which demonstrated that PM_2.5_ can exert cell damage [43]. However, the potential for any PM to produce adverse health effects is determined by their physical and chemical properties [43]. PAHs and metals are major contributors to toxicity of wood smoke particles. PAHS could only be detected in a rather low amount (unpublished results) and metals were under the detection limit, but PM_2.5_ consist of a high amount of inorganic salts which may explain their missing acute cytotoxicity. Nevertheless, activation of p38 MAPK or RhoA has been associated with endothelial or epithelial barrier disruption, characteristic of COPD or acute lung injury [44–46]. It is of note that these diseases are connected to high PM concentrations.

In an intermediate phase, prolonged PM_2.5_ exposure led to reduced proliferation, which was accompanied by both a G1 and G2/M phase arrest and translational impairment. Concomitant G1 and G2/M phase arrest has been described previously in primary lung fibroblasts exposed to extracts from cigarette smoke [47]. In our studies, eEF2—a strong indicator of global translational repression—was phosphorylated by an AMPK-dependent mechanism, which may result in reduced cyclin synthesis and explain the inhibition of cell-cycle progression at multiple restriction points [14, 21]. Our results further demonstrate both p38 MAPK activation and p21 CIP1/WAF1 up-regulation, which may negatively control cell-cycle progression during the G1 and the G2/M phases [23, 48, 49]. Transcriptional induction of p21 CIP1/WAF1 was independent of p53, because it is inactivated by the SV40 large T-antigen in the immortalized BEAS-2B cells. Therefore, we could neither observe stabilization, nor nuclear accumulation nor serine 392 phosphorylation of p53 in PM_2.5_-exposed cells (R. Dornhof, unpublished observations). Consistent with these results, diesel particles were also reported to upregulate p21 CIP1/WAF1 by a p53-independent mechanism [50].

In addition to p38 MAPK, AMPK was also activated in continuously exposed BEAS-2B cells. AMPK initiates autophagy by direct phosphorylation of ULK1, whereas p38 MAPK induces autophagy via beclin-1 synthesis upon activation of the transcriptional function of p53 [25, 33, 36]. In contrast to ULK1 phosphorylation, beclin-1 synthesis was not upregulated in the BEAS-2B cells exposed to PM_2.5_, because p53 is inactivated by the SV40 large T-antigen in these cells. A crosstalk between p38 MAPK and AMPK seems to be absent in BEAS-2B cells because pharmacologic inhibition of AMPK does not significantly influence p38 MAPK activation and *vice versa* (R. Dornhof, unpublished observations).

Our results indicate that, upon continuous exposure to PM, autophagy initially may represent a cytoprotective mechanism to save restricted energy sources during the intermediate stress response, which also involves AMPK. However, finally autophagy may contribute to cell death, because its inhibition led to restoration of cellular viability. Likewise, lung epithelial cells exposed to cigarette smoke or traffic- and industry-related particles displayed enhanced autophagy, caspase-3 activation and apoptotic cell death, which could be prevented by inhibition of autophagic proteins [51, 52]. Similarly, we also observed autophagy-related cell death in the presence of caspase-3 activity. However, apoptosis is only partly responsible for PM_2.5_-mediated cell death, because Q-VD-OPh, a pan-caspase inhibitor that protects cells from caspase-dependent apoptosis, only partly restored the viability of cells that were persistently treated with PM_2.5_.

Moreover, formation of autophagosomes during autophagy is also reported as an attempt to degrade foreign material [34]. Here, we provide evidence that PM_2.5_ from biomass combustion is internalized by BEAS-2B cells, stored in membrane-bound vesicles and fused with autophagosomes and lysosomes with the intention to degrade this foreign material. Failure of degradation seems to provoke the cell to continue with the fusion process, resulting in complex fusion products. This phenomenon has already been described for silica nanoparticles [37]. Lysosomal dysfunction has also been associated with several diseases characterized by lysosomal storage disorders [53], pointing to the importance of lysosomal degradation pathways to maintain cellular homeostasis [34]. Overloading of these complex fusion vesicles with inorganic particles resistant to lysosomal degradation can lead to perforation and rupture of the membranes [34, 37]. Partial lysosomal rupture by inorganic particles has been associated with the induction of the inflammasome and caspase-1 [27, 28], which might ultimately trigger cell death by pyroptosis. However, prolonged treatment with PM_2.5_ resulted in cell death without procaspase-1 cleavage and IL-1β release, indicating that the inflammasome and pyroptosis are not involved and that complete lysosome rupture triggered a cathepsin-dependent necrotic cell death [38]. Accordingly, the use of inhibitors of cathepsins maintained viability in BEAS-2B cells.

Moreover, TEM analysis revealed swollen and morphologically changed mitochondria, which are not eliminated by autophagy. Internalized PM_2.5_ could attack mitochondria directly, as reported for silica particles [54]. The damaging effect of PM_2.5_ may lead to disturbance of the energy content of the cells, which is confirmed by the observed reduced metabolic activity in the MTT assay and by the activation of AMPK.

## Conclusion

In summary, our results contribute to the explanation of PM-associated diseases on the basis of molecular events. Our findings demonstrate that cells are able to incorporate PM_2.5_ and cope with them to a certain concentration threshold. Initial induction of an adaptive stress response, i.e. at the onset of exposure, might explain why human health is only rarely directly affected by a short exposure to high concentrations of PM, whereas continuous exposure is expected to be most deleterious. However, it cannot be excluded that morphological changes might already lead to a predisposition for diseases associated with endothelial barrier dysfunction of PM. Upon prolonged PM_2.5_ exposure, proliferative regression with impaired energy homeostasis might further promote the progression of PM-associated diseases, e.g. by preventing an adequate immune response or cellular regeneration. Moreover, lysosomal dysfunction results in the accumulation of non-metabolized substrates and PM_2.5_ in complex fusion vesicles. Overloading of these vesicles with PM_2.5_ leads to membrane rupture and release of lysosomal enzymes, events that can promote cell death and may be involved in the pathogenesis of human diseases associated to high PM concentrations [34, 55].

## Supporting information

S1 File Characterization of fine particulate matter (PM_2.5_). (PDF)

S1 Fig PM_2.5_ composition as determined by scanning electron microscopy, X-ray diffraction analysis and Rietveld refinement. Color-coded SEM image of PM_2.5_ (**A**). Particles displayed different shapes, sizes and chemical composition. Small particles were mainly composed of potassium sulfate (K_2_SO_4_) and potassium chloride (KCl), whereas larger particles mainly consisted of calcium carbonates (CaCO_3_, CaMg(CO_3_)_2_) calcium hydroxide (Ca(OH)_2_), silicates and free lime (CaO). Mineralogical composition of PM_2.5_; values given in wt% (**B**).
(PDF)

S2 Fig Continuous exposure to PM_2.5_ reduces proliferation of BEAS-2B cells. Cells were left untreated or were exposed to 100 μg/ml PM_2.5_ for 5 weeks. In the S-phase BrdU incorporation into replicating DNA was determined for 15 h during re-exposure to PM_2.5_ for 48h.
(PDF)

S3 Fig Representative TEM images of complex fusion vesicles containing PM_2.5_ at the surface of the cell membrane of BEAS-2B cells. Scale in nm.
(PDF)

S4 Fig Representative TEM images of the intermediate phase of BEAS-2B cells after long-term exposure to PM_2.5_ (100 μg/ml). (**A**) Membrane bound vesicles containing PM_2.5_. (**B**, **C**) Vesicle (amphisome) after fusion of a membrane-bound vesicle with PM_2.5_ and an autophagosome. White arrows indicate swollen mitochondria. (**D**, **E**) Complex fusion products with PM_2.5_. Scale in nm.
(PDF)
