# Supplementary material for: Stress fibers, autophagy and necrosis by persistent exposure to PM2.5 from biomass combustion
Source: PLoS One. 2017 Jul 3;12(7):e0180291. doi: 10.1371/journal.pone.0180291 (PMC5495337; doi:10.1371/journal.pone.0180291)
Supplement: S1 File — (PDF) [file pone.0180291.s001.pdf]

Characterization of fine particulate matter ($PM_{2.5}$)

Fraction ($PM_{2.5}$) was characterized by a field-emission scanning electron microscope, equipped with an energy-dispersive X-ray detector (Helios Nano Lab 650, FEI, Hillsboro, OR), as well as by XRD (Bruker AXS D8 Advance, Bruker AXS GmbH, Karlsruhe, Germany). The following parameters were used to generate the XRD patterns: Cu-anode ($K\alpha_1$), range 5-75° 2Θ, step-size 0.01°, and dwell time 2 seconds per step. Rietveld refinement for semi-quantitative phase analysis was accomplished by using DIFFRAC.SUITE TOPAS 4.2 (Bruker AXS GmbH). The amorphous content was determined after adding a 100% crystalline corundum standard to the sample.
