# Supplementary material for: Stress fibers, autophagy and necrosis by persistent exposure to PM2.5 from biomass combustion
Source: PLoS One. 2017 Jul 3;12(7):e0180291. doi: 10.1371/journal.pone.0180291 (PMC5495337; doi:10.1371/journal.pone.0180291)
Supplement: S1 Fig — Color-coded SEM image of PM2.5 (A). Particles displayed different shapes, sizes and chemical composition. Small particles were mainly composed of potassium sulfate (K2SO4) and potassium chloride (KCl), whereas larger particles mainly consisted of calcium carbonates (CaCO3, CaMg(CO3)2) calcium hydroxide (Ca(OH)2), silicates and free lime (CaO). Mineralogical composition of PM2.5; values given in wt% (B). (PDF) [file pone.0180291.s002.pdf]

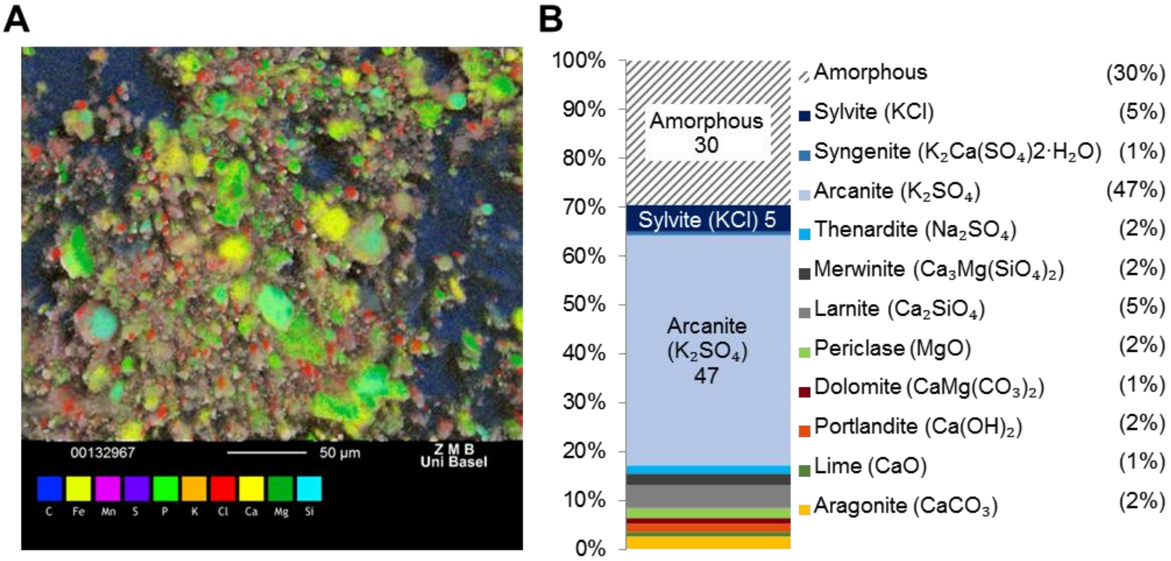

S1 Fig. $PM_{2.5}$ composition as determined by scanning electron microscopy, X-ray diffraction analysis and Rietveld refinement. Color-coded SEM image of $PM_{2.5}$ (**A**). Particles displayed different shapes, sizes and chemical composition. Small particles were mainly composed of potassium sulfate (K_2SO_4) and potassium chloride (KCl), whereas larger particles mainly consisted of calcium carbonates ($CaCO_3$, $CaMg(CO_3)_2$) calcium hydroxide ($Ca(OH)_2$), silicates and free lime (CaO). Mineralogical composition of $PM_{2.5}$; values given in wt% (**B**)
