# Supplementary material for: Stress fibers, autophagy and necrosis by persistent exposure to PM2.5 from biomass combustion
Source: PLoS One. 2017 Jul 3;12(7):e0180291. doi: 10.1371/journal.pone.0180291 (PMC5495337; doi:10.1371/journal.pone.0180291)
Supplement: S2 Fig — In the S-phase BrdU incorporation into replicating DNA was determined for 15 h during re-exposure to PM2.5 for 48h. (PDF) [file pone.0180291.s003.pdf]

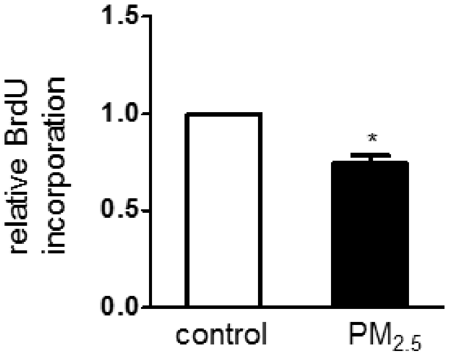

S2 Fig. Continuous exposure to $PM_{2.5}$ reduces proliferation of BEAS-2B cells. Cells were left untreated or were exposed to 100 µg/ml $PM_{2.5}$ for 5 weeks. In the S-phase BrdU incorporation into replicating DNA was determined for 15 h during re-exposure to $PM_{2.5}$ for 48h
