# Supplementary material for: Stress fibers, autophagy and necrosis by persistent exposure to PM2.5 from biomass combustion
Source: PLoS One. 2017 Jul 3;12(7):e0180291. doi: 10.1371/journal.pone.0180291 (PMC5495337; doi:10.1371/journal.pone.0180291)
Supplement: S3 Fig — Scale in nm. (PDF) [file pone.0180291.s004.pdf]

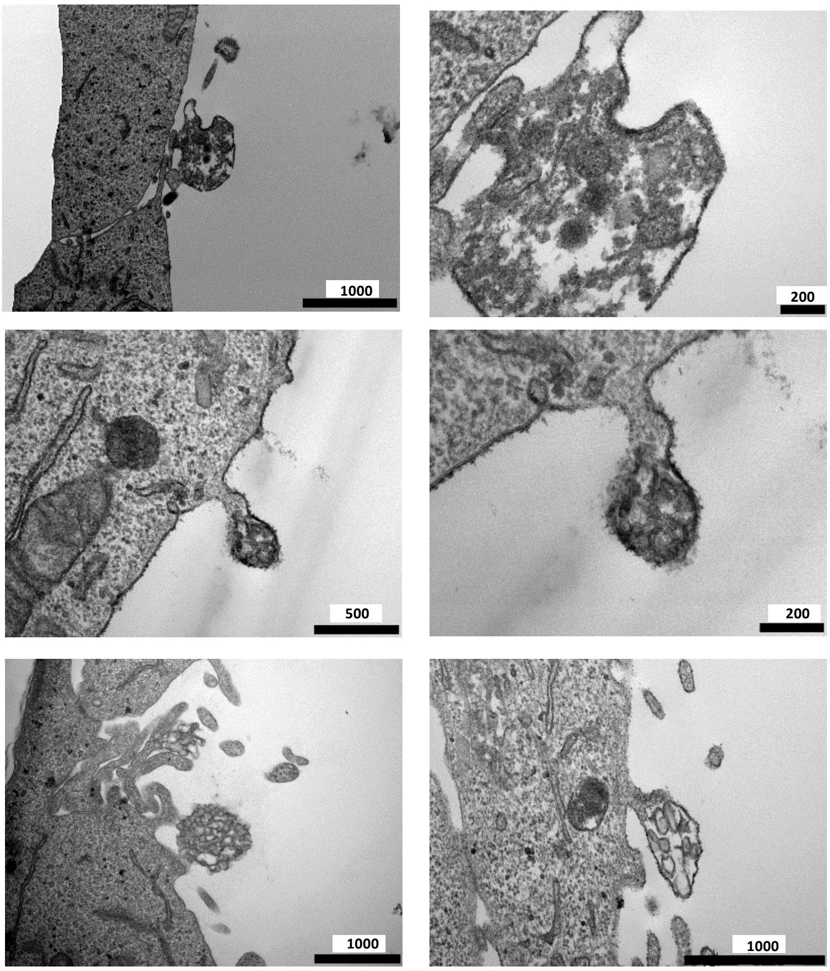

S3 Fig. Representative TEM images of complex fusion vesicles containing $PM_{2.5}$ at the surface of the cell membrane of BEAS-2B cells. Scale in nm
