# Supplementary material for: Stress fibers, autophagy and necrosis by persistent exposure to PM2.5 from biomass combustion
Source: PLoS One. 2017 Jul 3;12(7):e0180291. doi: 10.1371/journal.pone.0180291 (PMC5495337; doi:10.1371/journal.pone.0180291)
Supplement: S4 Fig — (A) Membrane bound vesicles containing PM2.5. (B, C) Vesicle (amphisome) after fusion of a membrane-bound vesicle with PM2.5 and an autophagosome. White arrows indicate swollen mitochondria. (D, E) Complex fusion products with PM2.5. Scale in nm. (PDF) [file pone.0180291.s005.pdf]

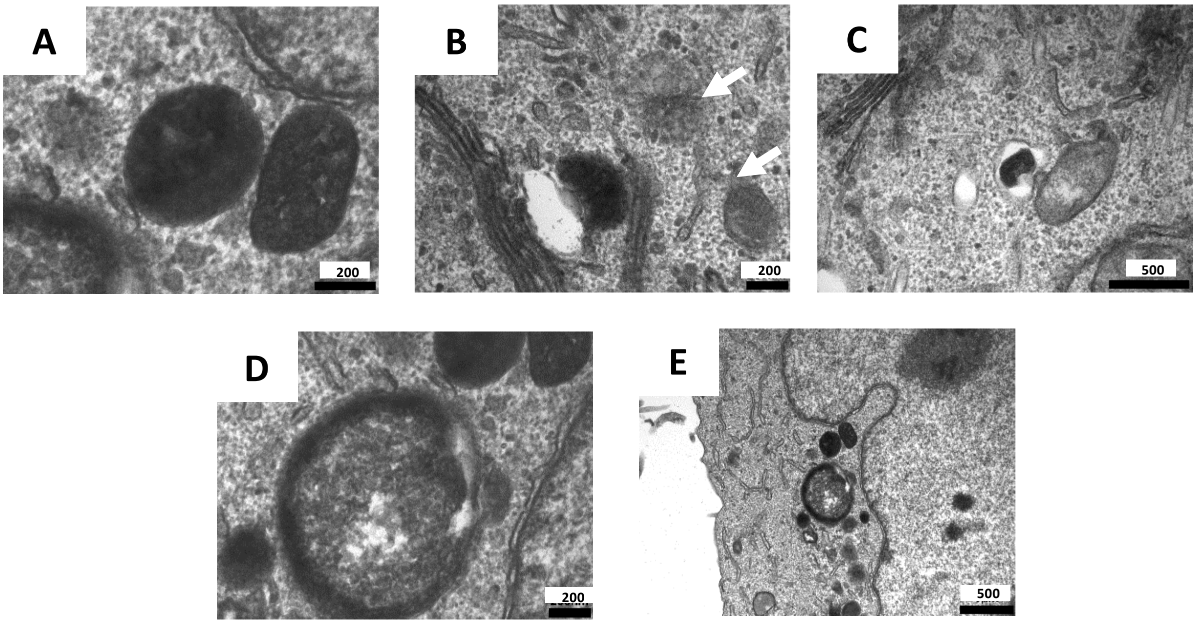

S4 Fig. Representative TEM images of the intermediate phase of BEAS-2B cells after long-term exposure to $PM_{2.5}$ (100 µg/ml). (**A**) Membrane bound vesicles containing $PM_{2.5}$. (**B**, **C**) Vesicle (amphisome) after fusion of a membrane-bound vesicle with $PM_{2.5}$ and an autophagosome. White arrows indicate swollen mitochondria. (**D**, **E**) Complex fusion products with $PM_{2.5}$. Scale in nm
